# Amino Acid PET – An Imaging Option to Identify Treatment Response, Posttherapeutic Effects, and Tumor Recurrence?

**DOI:** 10.3389/fneur.2016.00120

**Published:** 2016-07-28

**Authors:** Norbert Galldiks, Karl-Josef Langen

**Affiliations:** ^1^Department of Neurology, University of Cologne, Cologne, Germany; ^2^Institute of Neuroscience and Medicine, Forschungszentrum Jülich, Jülich, Germany; ^3^Center of Integrated Oncology (CIO), University of Cologne, Cologne, Germany; ^4^Department of Nuclear Medicine, University of Aachen, Aachen, Germany

**Keywords:** FET PET, MET PET, FDOPA PET, radiolabeled amino acids, pseudoprogression, pseudoresponse, radiation necrosis

## Abstract

Routine diagnostics and treatment monitoring in patients with primary and secondary brain tumors is usually based on contrast-enhanced standard MRI. However, the capacity of standard MRI to differentiate neoplastic tissue from non-specific posttreatment effects may be limited particularly after therapeutic interventions such as radio- and/or chemotherapy or newer treatment options, e.g., immune therapy. Metabolic imaging using PET may provide relevant additional information on tumor metabolism, which allows a more accurate diagnosis especially in clinically equivocal situations, particularly when radiolabeled amino acids are used. Amino acid PET allows a sensitive monitoring of a response to various treatment options, the early detection of tumor recurrence, and an improved differentiation of tumor recurrence from posttherapeutic effects. In the past, this method had only limited availability due to the use of PET tracers with a short half-life, e.g., C-11. In recent years, however, novel amino acid PET tracers labeled with positron emitters with a longer half-life (F-18) have been developed and clinically validated, which allow a more efficient and cost-effective application. These developments and the well-documented diagnostic performance of PET using radiolabeled amino acids suggest that its application continues to spread and that this technique may be available as a routine diagnostic tool for several indications in the field of neuro-oncology.

## Introduction

To date, structural MRI is the most important diagnostic tool in patients with brain tumors (1). Since decades, changes in contrast enhancement extent on MRI are used as an indicator of response to a certain therapy or tumor relapse (2, 3), although the reliability in distinguishing tumor tissue from treatment effects such as a blood–brain barrier breakdown is limited (4). For instance, reactive transient blood–brain barrier alterations with consecutive contrast enhancement – typically after radiotherapy with or without concomitant temozolomide – can mimic tumor progression and occur very early within the first 12 weeks after radiotherapy and is called pseudoprogression. Clinically, pseudoprogression is of substantial importance in neuro-oncology and occurs approximately in 10–30% of patients with malignant glioma (5–7). Other important side effects of radiotherapy are radiation-induced changes with late onset, particularly radiation necrosis, which usually manifests several months or even years later than pseudoprogression (8). Furthermore, transient blood–brain barrier breakdown may also result from cerebral ischemia, postoperative inflammation, or epileptic seizures.

After the approval of antiangiogenic drugs (e.g., bevacizumab), the so-called phenomenon “pseudoresponse” has been described, which may complicate the assessment of treatment response by evaluating changes in contrast enhancement according to the Macdonald criteria only (3). Within a few weeks after the initiation of treatment, it has been observed that antiangiogenic drugs, such as bevacizumab, are able to markedly reduce contrast enhancement (9), producing response rates consistent with partial or even complete responses. Some of these partial or complete responses observed on MR images may result from a quick normalization of abnormally permeable blood vessels, indicating – at least in part – a restoration of the blood–brain barrier integrity. Thus, a decrease in contrast enhancement, which indicates a response on MRI, may not reflect true antitumoral effects of antiangiogenic drugs (10). Furthermore, following antiangiogenic treatment, MRI suggests, not infrequently, an impressive radiological response, which can be in clear contrast to the clinical benefit caused by antiangiogenic therapy effects. Hence, the use of antiangiogenic drugs, most probably, affects the image findings of enhancing tumor portions considerably, more effectively than that of non-enhancing parts of the tumor (10). In order to overcome the limitations of the assessment of tumor response to antiangiogenic treatment by evaluation of changes in contrast enhancement only (according to the Macdonald criteria), the Response Assessment in Neuro-Oncology (RANO) group suggested new recommendations for evaluating response (2). Particularly, for antiangiogenic drugs, FLAIR or T2 signal hyperintensity was recommended as a surrogate marker for non-enhancing tumor to help determine tumor progression, and thereby include non-enhancing FLAIR or T2 signal alterations as criteria for determining tumor response or progression (“non-enhancing tumor progression”) (2).

However, present RANO criteria do not provide quantitative values of FLAIR or T2 signal change for the diagnosis of tumor progression. Various differential diagnoses, such as tumor-related edema, radiation injury, demyelination, ischemia, and infection, can result in a hyperintense FLAIR or T2 signal alteration, which is difficult to distinguish from non-enhancing tumor (10). Consequently, alternative diagnostic methods are necessary to improve the identification of treatment response, posttherapeutic effects, and tumor recurrence.

## Treatment Response

The feasibility and usefulness of amino acid PET using the tracers ^11^C-methyl-l-methionine (MET), *O*-(2-[^18^F]fluoroethyl)-l-tyrosine (FET), and 3,4-dihydroxy-6-[^18^F]-fluoro-l-phenylalanine (FDOPA) for treatment assessment after radiochemotherapy, stereotactic brachytherapy, alkylating chemotherapy, and antiangiogenic therapy using bevacizumab and other experimental approaches have been demonstrated in several studies and case series. The currently available PET data regarding these tracers suggest that both a reduction of amino acid uptake and a decrease of the metabolically active tumor volume of a glioma are a sign of response to treatment.

### Radiotherapy

A prospective study evaluated the prognostic value of early changes of FET uptake after postoperative radiochemotherapy in patients with glioblastoma (11, 12). It could be observed that PET responders with a reduction of the maximal tumor/brain ratio at least of more than 10% had a significantly longer progression-free and overall survival than patients with stable or increasing tracer uptake after radiochemotherapy. Regarding stereotactic brachytherapy using iodine-125 seeds, both tumor/brain ratios and metabolically active tumor volumes as determined by FET PET were able to differentiate between late posttherapeutic effects after 6 months or later and local tumor progression with high diagnostic accuracy (13).

### Alkylating Chemotherapy

A reliable monitoring of alkylating chemotherapy, i.e., temozolomide and nitrosoureas such as lomustine, could be observed with MET PET in recurrent or progressive high-grade glioma patients (14–16) and both with FET and MET PET in patients with recurrent or progressive low-grade glioma (17–19). Particularly, response to treatment in low-grade gliomas was associated with an earlier reduction of the FET PET tumor volume when compared to FLAIR/T2 signal changes.

### Antiangiogenic Therapy

More recent studies suggest that especially the decrease of the metabolically active tumor volume as assessed by amino acid PET using the tracers FET and FDOPA is useful to assess antiangiogenic therapy failure of bevacizumab earlier than MRI according to RANO criteria and, moreover, to identify responders to bevacizumab with favorable outcome (20–23).

In a series of 11 patients, FET PET detected failure of antiangiogenic therapy in 4 patients earlier than standard MRI (22). Similar results were observed in another study including 10 patients (21). In this study, treatment response based on RANO criteria was discordant in four patients to FET PET findings, indicating pseudoresponse on MRI. Furthermore, FET PET was able to detect tumor progression earlier than MRI (median time benefit, 10.5 weeks). In these FET PET studies (21, 22), a favorable outcome of responders to bevacizumab was observed when a decrease of the metabolically active tumor volume of 45% or more was present. Furthermore, a cost-effectiveness analysis suggests that the additional use of FET PET in the management of patients with recurrent high-grade glioma treated with bevacizumab may be cost-effective (24).

Using FDOPA PET, a recent study including 30 patients reported that responders based on FDOPA PET data survived 3.5 times longer than non-responders. In contrast, responders based on RANO criteria lived only 1.5 times longer than non-responders (20). Furthermore, this study also demonstrated that changes in the metabolically active tumor volume were highly prognostic. The absolute metabolically active tumor volume at the first follow-up scan (threshold, 18 ml) provided the strongest prediction of progression-free and overall survival (20).

### Other Treatment Options

In various experimental treatment options such as intracavitary radioimmunotherapy, convection-enhanced delivery of paclitaxel, and adjuvant maintenance therapy with imatinib in combination with hydroxyurea (25–27) treatment effects could be successfully monitored by PET using MET and FET.

## Posttherapeutic Effects and Tumor Recurrence

**Figure 1:**
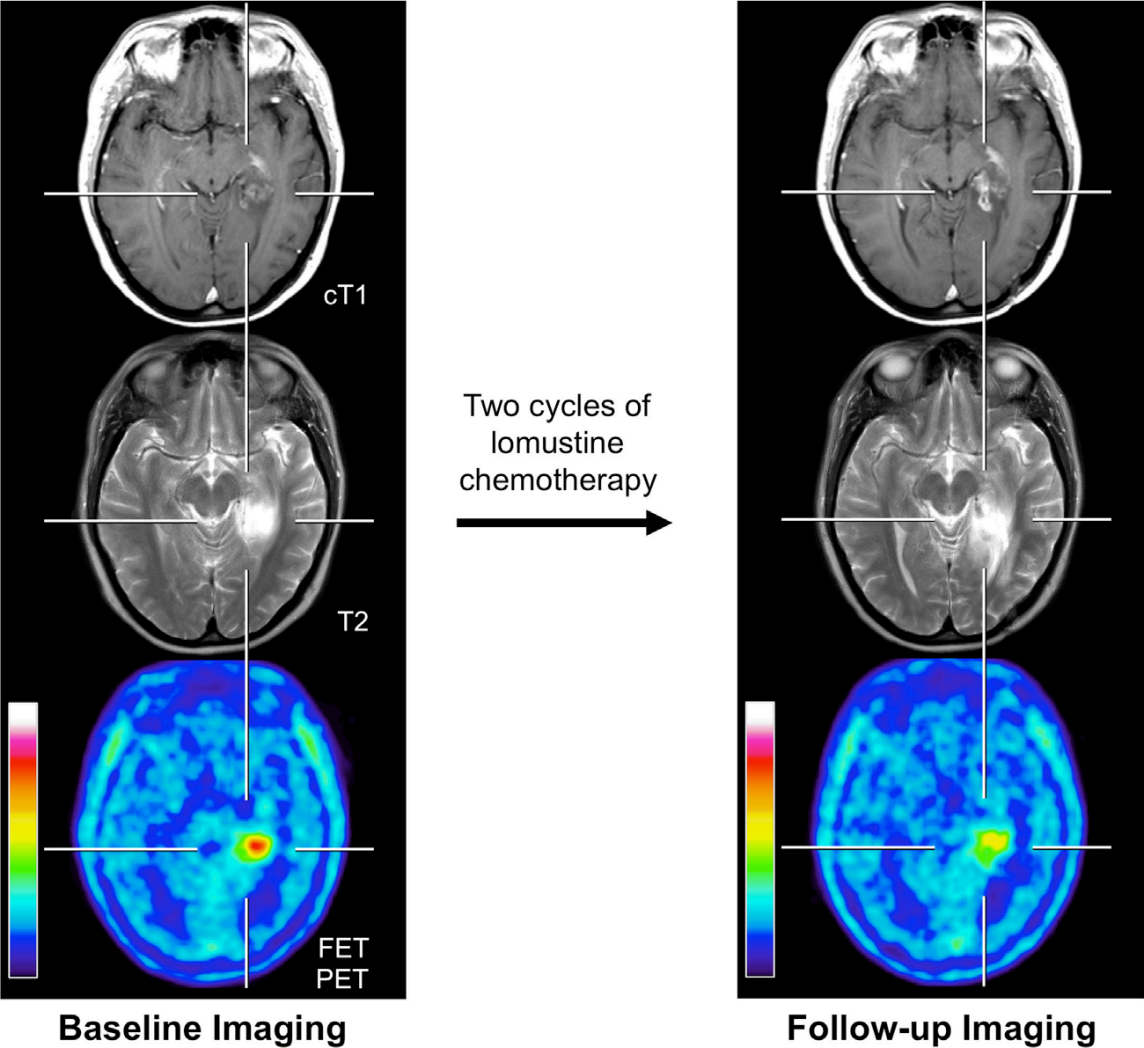
**Example of a 31-year-old patient with an anaplastic astrocytoma in the left parahippocampal region with posttherapeutic effects**. Previous treatment consisted brachytherapy and external fractionated radiotherapy with concomitant and adjuvant temozolomide. Brain imaging with standard MRI and FET PET at recurrence/before initiation of chemotherapy (left column) and 4 months later after two cycles of lomustine chemotherapy (right column). Enlargement of contrast enhancement and the T2 signal is suggesting tumor progression, whereas FET PET indicates a reduction of metabolic activity.

After neuro-oncological treatment, particularly, non-neoplastic increase in contrast enhancement (Figure 1) or a newly diagnosed contrast-enhancing lesion on standard MRI have been found, which may considerably confuse patient management in neuro-oncology because posttherapeutic effects cannot be always ruled out. In this setting, the value of amino acid PET using the tracers MET, FET, and FDOPA for the identification of tumor recurrence or progression in patients with low-grade and high-grade glioma has been described in many studies (28–35). Overall, a higher diagnostic performance compared to standard MRI has been observed. However, in many of these studies, early delayed posttherapeutic effects were not further specified from late effects.

### Pseudoprogression

**Figure 2:**
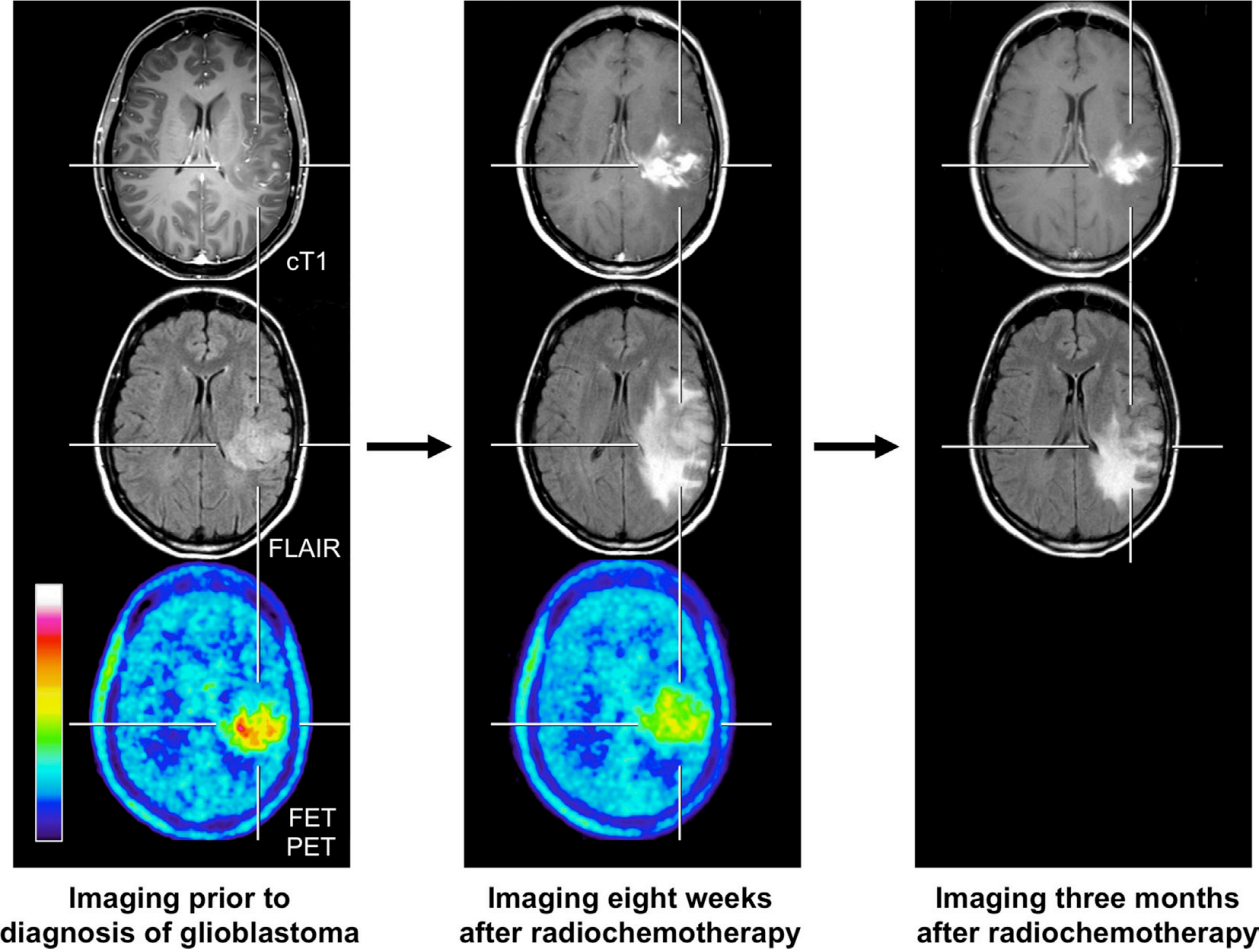
**FET PET and conventional MR imaging in a 47-year-old patient prior to histological confirmation of glioblastoma diagnosis (left column), and 8 weeks (middle column) and 3 months after completion of radiochemotherapy with temozolomide (right column)**. The follow-up MR images 8 weeks after completion of radiochemotherapy suggests markedly tumor progression (middle column). In contrast, the FET PET image shows decreased metabolic activity compared to initial FET PET (maximum tumor/brain ratio 3.3 vs. 4.7) indicating pseudoprogression. Correspondingly, follow-up MRI, 3 months after radiochemotherapy, shows an improvement with regressive findings without change in the treatment regimen (right column).

After the implementation of chemoradiation with concurrent temozolomide for glioblastoma patients representing the current standard of care, there has been an increasing awareness of progressive contrast-enhancing lesions on conventional MRI, which are not related to a true disease progression, but which are most probably due to a treatment effect and are called pseudoprogression. This phenomenon occurs usually within the first 12 weeks after chemoradiation with concurrent temozolomide or radiotherapy alone (2, 5, 36), and this time, frame has been implemented into the RANO criteria (2). If histology is not available, pseudoprogression is usually retrospectively diagnosed and is based on increasing contrast enhancement on MRI suggesting tumor progression that eventually remains stable or even regresses during further follow-up MRI imaging without any change in treatment (37) (Figure 2). However, cases with a later onset of pseudoprogression have been observed, particularly after chemoradiation, using temozolomide in combination with nitrosoureas such as lomustine (CCNU) (38, 39). In high-grade glioma patients, the rate of pseudoprogression seems to be between 10 and 30% (5–7) and is clinically of great importance because an effective therapy might be erroneously terminated, potentially and negatively impacting the outcome.

Unfortunately, standard MR imaging does not allow a reliable differentiation of tumor relapse from pseudoprogression. Several studies have suggested that, within a narrow time frame of 12 weeks after completion of radiochemotherapy, FET PET might be helpful for this distinction (11, 40, 41). Subsequently, in a larger patient cohort with glioblastomas (*n* = 22), the diagnostic accuracy for FET PET of more than 90% for differentiating pseudoprogression from true tumor progression after chemoradiation with concurrent temozolomide has been observed (42). In line with this study, FDOPA PET may also be useful for diagnosing patients with pseudoprogression. A study with glioblastoma patients (*n* = 110) revealed an accuracy of 82% for FDOPA PET for the correct diagnosis of true tumor progression or recurrence (32). However, in this study, early and late delayed treatment-induced changes, such as pseudoprogression or radiation necrosis, were not further specified. Thus, despite the lack of subsequent PET studies with a higher number of patients evaluating this particular time window of 12 weeks after chemoradiation, the present data on amino acid PET suggest that this technique is helpful for the diagnosis of pseudoprogression following chemoradiation of glioblastoma.

### Radiation Necrosis

This phenomenon is an important side effect of radiotherapy and belongs to radiation-induced changes with late onset and usually manifests several months or even years later than pseudoprogression (8). A radiation necrosis may occur after radiotherapy of a glioma as well as brain metastasis.

In view of both the sociodemographic changes with an increasing elderly population and a wider spectrum of treatment options for extracranial tumors resulting in an improvement of outcome, an increasing number of patients diagnosed with brain metastasis can be expected (43, 44). Besides neurosurgical resection, radiotherapy options, such as brachytherapy, radiosurgery, and whole-brain radiation therapy, are frequently used to treat patients with brain metastasis. In this group of patients, neuro-oncologists are often confronted with the clinical problem that after radiation therapy, and, in particular, after radiosurgery, standard MRI cannot reliably differentiate brain metastasis recurrence or progression from radiation necrosis.

Following radiosurgery in brain metastasis patients, a considerable rate of radiation necrosis (24% of 310 cerebral metastases) has been reported (45). Depending on the irradiated volume receiving a critical radiation dose, the risk of radiation necrosis may increase up to 47% (45). Moreover, some studies in patients with primary brain tumors suggest that radiation necrosis occurs in approximately 5–25% of patients receiving standard radiotherapy (4, 46).

In order to address this highly relevant clinical problem, amino acid PET has been recently used. For instance, simple semiquantitative regions-of-interest (ROI) analyses were used in MET PET studies for the calculation of tumor-to-brain ratios and revealed a sensitivity and specificity of 70–80% for the differentiation of local brain metastasis recurrence from radiation-related effects (47, 48). Similarly, a FDOPA PET study revealed a sensitivity and specificity of more than 80% (49). Another study has compared the diagnostic performance of FDOPA PET with that of perfusion-weighted MRI (PWI) in patients with brain metastases after stereotactic radiosurgery. In this study, the accuracy of FDOPA PET was 91% and superior to PWI metrics, which yielded an accuracy of 76% (50). In line with this, a similar diagnostic performance has also been observed for FET PET: using tumor/brain ratios in combination with the evaluation of time-activity curves derived from kinetic FET PET scans, a sensitivity and specificity of about 90% could be observed (51).

## Conclusion and Perspectives

In summary, the use of amino acid PET imaging is helpful to provide an early assessment of therapy efficacy and for the differentiation of posttherapeutic effects from tumor recurrence. This technique aids oncologists to optimize therapeutic management of brain tumors and has the potential to evaluate response to newer treatment options such as immune or targeted therapy.

Regarding the tracer selection it has to be considered that, in comparison to MET labeled with C-11 (half-life, 20 min), the main advantage of FET is the longer half-life of the F-18 label (110 min), which allows a widespread clinical distribution. In addition, FET uptake appears to be more specific for neoplastic tissue, because there is a higher uptake of MET in inflammatory cells and tissues. The lower specificity of MET may be explained by its higher affinity for macrophages compared with FET as demonstrated in animal experiments (52). Of great interest is the presence of differential FET uptake kinetics in malignant gliomas and low-grade gliomas, which have not been observed in other amino acid tracers (53, 54), and the lack of physiological uptake in the basal ganglia when compared with FDOPA PET (44). Hence, FET seems to be the most promising amino acid tracer for PET imaging in brain tumor patients and should be considered for prospective studies (55).

It has to be noted that amino acid PET tracers eventually may show uptake in non-tumoral brain lesions. In contrast to MET, experiments in animal models have shown that FET exhibits no uptake in inflammatory lymph nodes and in inflammatory cells. However, false positive tracer uptake has been observed for MET as well as for FET in patients with brain abscesses, demyelinating processes, hematomas, cerebral ischemia, or in cases with pronounced radionecrosis (56–58). Additionally, in glioma patients with seizure clusters or status epilepticus, a transient FET uptake in cortical brain areas has been observed, which were not affected by tumor tissue (44). Therefore, increased uptake of the tracers is not thoroughly specific for cerebral gliomas although increased amino acid uptake has a high positive predictive value for cerebral gliomas (59). In clinical practice, false positive uptake in non-tumoral lesions is rare (30, 42, 60) and usually mild, thus affecting the diagnostic performance of amino acid PET imaging insignificantly.

Furthermore, the diagnostic performance of amino acid PET needs to be compared with advanced MRI techniques, e.g., diffusion- and perfusion-weighted imaging, sodium MRI, and chemical exchange saturation transfer (CEST) imaging. There is evidence that these methods may be helpful to differentiate tumor relapse from posttherapeutic effects (61–63). In order to provide the optimal diagnostic work-up to the individual patients, multimodal imaging studies should be on the basis of reader-independent image evaluation. From these imaging studies, surrogate parameters need to be derived, which can then be used in clinical routine.

In order to identify these surrogate imaging markers, the use of hybrid PET/MR imaging technology may be helpful. This technique allows the simultaneous acquisition of valuable diagnostic information. For example, recent studies support the acquisition of dynamic FET PET, standard anatomical MRI sequences, and PWI MRI in a single session (“one-stop-shop”) on a hybrid PET/MR scanner in glioma patients (64, 65). Moreover, this technology helps to minimize the patients’ discomfort (e.g., considerable reduction of scanning time, only a single transport to the imaging facility, avoidance of sedation or anesthesia, particularly in children) and helps to optimize co-registration of various imaging modalities. However, the MRI-based attenuation correction can be very challenging.

As we move forward with new technologies and innovations in neuroimaging, the challenge of determining pseudoprogression from true tumor progression may be resolved. Combining the efforts and knowledge of interested researchers in this way will hasten the solution to this problem.

## Ethics Statement

The local ethics committee approved the evaluation of retrospectively collected patient data. All patients gave written-informed consent prior to each PET investigation.

## Author Contributions

NG: writing of the manuscript. K-JL: review of the manuscript.

## Conflict of Interest Statement

The authors declare that the research was conducted in the absence of any commercial or financial relationships that could be construed as a potential conflict of interest.
